# Comparison of the Superior and Inferior Rectus Muscles in Humans: An Anatomical Study with Notes on Morphology, Anatomical Variations, and Intramuscular Innervation Patterns

**DOI:** 10.1155/2020/9037693

**Published:** 2020-04-30

**Authors:** Robert Haładaj, Michał Polguj, R. Shane Tubbs

**Affiliations:** ^1^Department of Normal and Clinical Anatomy, Anatomy and Histology, Medical University of Lodz, Poland; ^2^Department of Neurosurgery, Tulane Center for Clinical Neurosciences, Tulane University School of Medicine, New Orleans, LA, USA; ^3^Department of Anatomical Sciences, St. George's University, St. George's, Grenada

## Abstract

A comparison of the superior and inferior rectus muscles was performed to determine whether they have similar structures and innervation attributable to their participation in the same type of, although antagonistic, eye movements. The study was conducted on 70 cadaveric hemiheads, and the anatomical variations in the superior and inferior rectus muscles were assessed. Sihler's whole mount nerve staining technique was used on 20 isolated superior and 20 isolated inferior rectus muscle specimens to visualize the intramuscular distribution of the oculomotor nerve subbranches. In two cases (~2.8%), variant muscular slips were found that connected the superior and inferior rectus muscles. In 80% of cases, muscular branches arising directly from the inferior branch of the oculomotor nerve innervated the inferior rectus muscle, while in 20% of cases, the nerve to the inferior oblique muscle pierced the inferior rectus muscle and provided its innervation. In 15 of 70 specimens (21.4%), a branch to the levator palpebrae superioris muscle pierced the superior rectus muscle. The distance between the specific rectus muscle's insertion and the anterior-most terminations of the nerves' subbranches with reference to the muscle's total length ranged from 26.9% to 47.2% for the inferior rectus and from 34.8% to 46.6% for the superior rectus, respectively. The superior rectus muscle is slightly longer and its insertion is farther from the limbus of the cornea than is the inferior rectus muscle. Both muscles share a common general pattern of intramuscular nerve subbranches' arborization, with characteristic Y-shaped ramifications that form the terminal nerve plexus located near half of the muscles' length. Unexpected anatomical variations of the extraocular muscles may be relevant during orbital imaging or surgical procedures.

## 1. Introduction

The four extraocular rectus muscles originate from the common tendinous ring and extend forward to attach to the eyeball by tendinous expansions that insert into the sclera [1, 2]. The superior rectus muscle is longer than other rectus muscles [3] and originates superior and lateral to the optic nerve. The origin of the inferior rectus muscle is located below the optic nerve. Both the superior and inferior rectus muscles are thickest at their midlength and gradually become thinner at their distal ends [3]. The superior rectus directs the eye upward (elevates it), while the inferior rectus directs the eye downward (depresses it), and the two are each muscle's primary action, respectively. Because of their location and primary action, these muscles are referred to as “the vertical rectus muscles” [4–6].

Anatomical variations of the extraocular muscles are rare and, among others, may include occurrence of additional bellies, muscular slips, or bridges between selected muscles, as well as some differences in their nerves' course and distribution [7–10]. Such variations may alter the normal anatomical relations of structures located within the orbit, which may be of particular importance in orbital imaging or surgeries performed on the extraocular muscles.

A thorough analysis of the literature revealed that only a few papers include detailed studies of the extraocular muscles' intramuscular innervation carried out on human specimens. Moreover, to date, these studies have focused primarily on the horizontal (lateral and medial) rectus muscles [11–14]. Therefore, we applied Sihler's whole mount nerve staining technique to improve evaluation of the nerves' distribution within the vertical rectus muscles. Sihler's stain is a gross anatomical technique that allows tracing the detailed intramuscular course and branching pattern of nerves [15, 16]. Based on animal tissues, Gözil et al. [17] and Gülekon et al. [18] suggested that Sihler's stain technique may be useful for detailed studies of extraocular muscles' motor control and may improve our understanding of those muscles' structure and function. Detailed knowledge of the nerves' distribution patterns and possible aberrations in their course also may increase the safety and success rate of surgical procedures performed within the orbit and on the extraocular muscles themselves [13, 14], as keeping the innervation intact maintains the muscle's function.

In this context, the goal of our study was to supplement earlier findings by examining morphology, anatomical variations, and detailed patterns of intramuscular nerves' distribution within the vertical (superior and inferior) rectus muscles. It also is one of the first studies that uses Sihler's staining technique in research on human superior and inferior rectus muscles. Using Sihler's stain with regard to the superior and inferior rectus muscles is a novelty of the study. The comparison of both muscles was performed to determine whether there are similarities in structure and innervation attributable to participation in the same type of, although antagonistic, eye movements. The paper provides a detailed anatomical investigation of the superior and inferior rectus muscles in the human and includes their variations (atypical muscular bridges that link the muscles) as well. A discussion of the clinical significance of the presented findings also is included.

## 2. Materials and Methods

First, we obtained the local bioethics committee's approval for the study, which was conducted on 70 (32 left and 38 right) cadaveric hemiheads. The bony dissections were performed using protocols described previously [19–23]. The roof and a large part of the orbit's lateral wall were removed on both sides using a Luer bone rongeur and bone chisel. The superior orbital fissure and the optic canal were also opened, after which the orbit's content was harvested *en bloc*.

After the superior and inferior rectus muscles were visualized over their entire course, detailed measurements were performed, including the muscles' length (measured between their origin and insertion), width (at the level of the origin, midlength, and insertion), thickness at the midlength, distance between the limbus and anterior-most end of the muscles' insertion, and the distance at which the oculomotor nerve's branches reached the muscles' internal surface (measured from the anterior-most end of their insertion to the anterior-most muscular branches that reach the internal muscles' surface). To compare measurements taken from individual specimens, the distance between the insertion and the point where the oculomotor nerve's subbranches reached the muscles' internal surface was referenced (as a quotient) to their total length, and basic descriptive statistics were applied to the raw data.

The superior and inferior rectus muscles' morphology, including their anatomical variations (i.e., presence of additional vertical muscular slips that link the two muscles), was assessed. Cases of accessory muscular slips were subjected to histological examination. The classical paraffin method was used to obtain serial sections (each approximately 7 *μ*m thick). Then, standard hematoxylin and eosin (H&E) staining was performed. The samples obtained through this procedure were evaluated under a biological microscope with an installed camera.

To visualize the oculomotor nerve subbranches' intramuscular distribution, 20 isolated superior and 20 isolated inferior rectus muscle specimens were stained using Sihler's whole mount nerve staining technique, according to the procedure described by Mu and Sanders [15], Won et al. [16], and Shin et al. [13, 14], with slight modifications (only newly fixed specimens were subjected to Sihler's stain). This technique allowed the muscles' detailed intramuscular innervation pattern to be visualized. Based on our earlier experience, the concentration of individual chemicals was adjusted for a small muscle mass [21–23]. During the procedure's first stage, maceration and depigmentation, isolated muscles were immersed in a reduced concentration of KOH solution (2.5% versus 3% according to the original recipe) [21–23]. This initial phase of Sihler's stain required 3 to 5 weeks. The next stage, decalcification, was performed with standard “Sihler's solution I” [15, 16]. This stage took approximately 1 week. Whole mount nerve staining was performed using standard “Sihler's solution II” [15, 16]; this stage took up to two weeks. During the last stage (destaining), a lower concentration of acetic acid in “Sihler's solution I” was used to control the destaining process better (0.5 : 1.5 : 6 = glacial acetic acid : glycerin : 1%aqueous chloral hydrate) [21–23]. The course of each stage varied among individual specimens depending on the sample's quality. Based on Sihler's stain, the location of the foremost “tufty” terminations was assessed and expressed as a percent of the total muscle's length beginning from its origin.

## 3. Results

### 3.1. Superior and Inferior Rectus Muscles' Major Anatomical Variations

**Figure 1:**
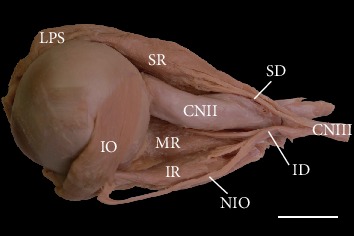
Typical appearance of the superior (SR) and inferior (IR) rectus muscles. Lateral view of the specimen harvested from the left orbit (the lateral rectus muscle has been removed). Typical distribution and territory of the oculomotor nerve's (CNIII) branches (the ciliary ganglion with its parasympathetic root has been removed). The nerve to the inferior oblique (NIO) muscle runs along the inferior rectus muscle's lateral border. Scale bar = 10 mm. CNII: optic nerve; SD: superior branch of the oculomotor nerve; ID: inferior branch of the oculomotor nerve; IO: inferior oblique muscle; LPS: levator palpebrae superioris muscle; MR: medial rectus muscle.

In all cases examined, the inferior and superior rectus muscles' origin was located in a fibrous ring referred to as the common tendinous ring. The inferior rectus' insertion was attached to the eyeball's anteroinferior surface, while the superior rectus muscle's insertion was located on its anterosuperior surface (both muscles' insertions were located anterior to the equator of the eyeball). The mean distance measured from the limbus to the inferior rectus muscle's insertion was 6.2 mm (min = 5.4 mm, max = 6.9 mm, median = 6.2 mm, and SD = 0.5 mm). The mean distance measured from the limbus to the superior rectus' insertion was 7.6 mm (min = 6.4 mm, max = 8.7 mm, median = 7.6 mm, and SD = 0.8 mm). The vertical rectus muscle's additional detailed morphometric characteristics are presented in Tables 1 and 2. The typical anatomy of both vertical rectus muscles is presented in Figure 1.

**Figure 2:**
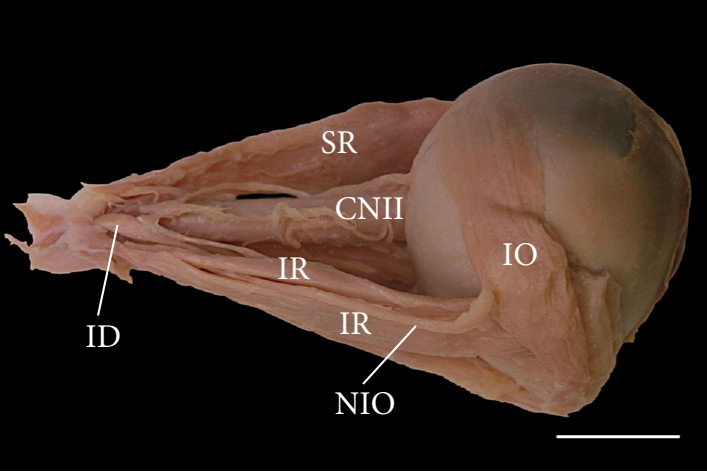
Inferior rectus (IR) muscle pierced by the nerve to the inferior oblique (NIO) muscle. Lateral view of the specimen harvested from the right orbit (the lateral rectus muscle has been removed). Scale bar = 10 mm. CNII: optic nerve; ID: inferior branch of the oculomotor nerve; IO: inferior oblique muscle; SR: superior rectus muscle.

**Figure 3:**
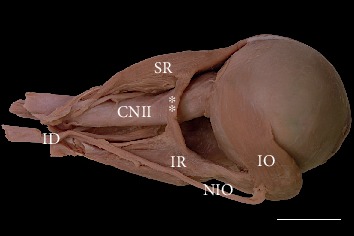
Variant muscular slip (marked as double white asterisks) connecting the superior and inferior rectus muscles. Lateral view of the specimen harvested from the right orbit (the lateral rectus muscle has been removed). An atypical muscular bridge between the superior (SR) and inferior (IR) rectus muscles occupies the position lateral to the optic nerve (CNII). Scale bar = 10 mm. ID: inferior branch of the oculomotor nerve; IO: inferior oblique muscle; NIO: nerve to the inferior oblique muscle.

The inferior rectus muscle's most common anatomical variation included cases with atypical topographical relations between the muscle and nerve to the inferior oblique muscle. Typically, the nerve to the inferior oblique muscle ran along the muscle's lateral border (Figure 1). However, in 14 of 70 cases (20%), this nerve pierced the inferior rectus. In those cases, the nerve to the inferior oblique entered the inferior rectus muscle's fibers just after originating from the oculomotor nerve end's inferior branch and emerged from among the muscle's fibers in the proximal half of its length (Figures 2 and 3).

**Figure 4:**
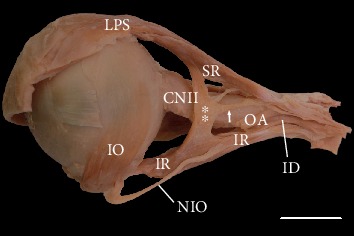
Variant muscular slip (marked as double white asterisks) connecting the superior and inferior rectus muscles. Lateral view of the specimen harvested from the left orbit (the lateral rectus muscle has been removed). In this case, a tendinous band (marked as a white arrow) arose from the muscular bridge and attached to the common tendinous ring. An atypical muscular connection between the superior (SR) and inferior (IR) rectus muscles occupies the position lateral to the optic nerve (CNII). The nerve to the inferior oblique (NIO) muscle pierces the inferior rectus muscle. Scale bar = 10 mm. ID: inferior branch of the oculomotor nerve; IO: inferior oblique muscle; LPS: levator palpebrae superioris muscle, OA: ophthalmic artery (cut).

**Figure 5:**
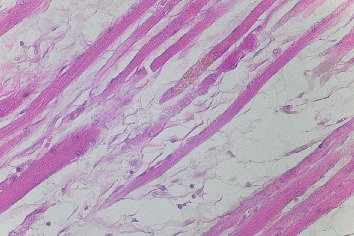
Sample fibers of striated skeletal muscle obtained from the muscular bridge linking the superior and inferior rectus muscles (taken from the specimen shown in Figure 3). The skeletal muscle tissue's striations are shown. H&E stain, ×40 objective.

In two cases (~2.8%), variant muscular slips that connected the superior and inferior rectus muscles were found (Figures 3 and 4). In one, an additional tendinous band arose from this muscular bridge and attached to the common tendinous ring (Figure 4). Both atypical muscular connections between the superior and inferior rectus muscles occupied a position lateral to the optic nerve. In one case, the ciliary ganglion was located just between the muscular slip and lateral rectus muscle. In the second case, three short ciliary nerves crossed the variant slip's lateral surface. Both specimens of muscular slips that connected the superior and inferior rectus muscles were subjected to histological examination, and the presence of striated skeletal muscle tissue was confirmed in both cases (Figure 5). No separate nerve branches to the muscular bridges were observed macroscopically during the dissection.

### 3.2. Vertical Extraocular Muscles' Innervation Pattern

**Figure 6:**
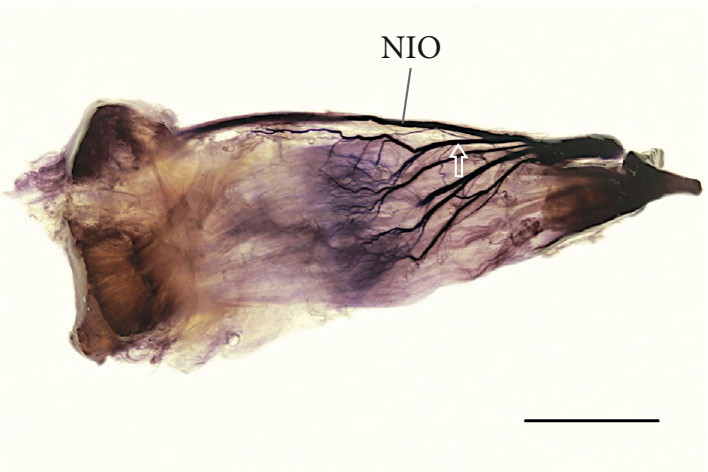
Intramuscular arborization of the oculomotor nerve's inferior branch within the human right inferior rectus muscle. View to the muscle's internal surface (surface facing the eyeball). Sihler's stain. Scale bar = 10 mm. Four primary subbranches are visible, one of which (marked by the white outline of the arrow) branches off the nerve to the inferior oblique (NIO) muscle as an anatomical variant. Subbranches running within the muscle formed a characteristic tree-like branching pattern. The terminal plexus with tufty endings of muscular subbranches is visible near the proximal half of the muscle.

In all specimens examined, the superior rectus muscle was innervated solely by muscular branches arising from the superior branch of the oculomotor nerve. In 56 of 70 cases (80%), muscular branches arising directly from the oculomotor nerve's inferior branch innervated the inferior rectus muscle. However, detailed examination using Sihler's stain revealed that occasionally (3/20; 15%), there were additional branches from the nerve to the inferior oblique muscle (Figure 6). In all 14 specimens in which the nerve to the inferior oblique muscle pierced the inferior rectus muscle, the muscular branches to the inferior rectus muscle arose from that nerve. The detailed morphometric characteristics of points where the muscular branches reached the inferior and superior rectus muscles' bellies are presented in Tables 1 and 2, respectively.

Considering the specimens examined using Sihler's stain, one to four primary muscular branches supplied the inferior rectus muscle (mean = 2.5, median = 2.5, and SD = 0.86). The diameters of the primary branches to the inferior rectus muscle ranged from 0.11 mm to 0.82 mm (mean = 0.29 mm, median = 0.26 mm, and SD = 0.17 mm). Sihler's stain allowed us to observe that from three to nine secondary subbranches reached the muscle's internal surface, i.e., surface facing the eyeball (mean = 6.2, median = 5.5, and SD = 1.9; see Figure 6). The distance at which the motor nerve subbranches penetrated the internal muscle's surface measured from the muscle's insertion ranged from 21.5 mm to 29.6 mm (Table 1). The same distance referenced (as a quotient) to the muscle's total length ranged from 49.4% to 71.3% (Table 1).

**Figure 7:**
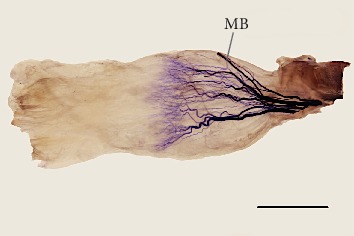
Intramuscular arborization of the superior branch of the oculomotor nerve within the human right superior rectus muscle. View to the muscle's internal (inferior) surface. Sihler's stain. Scale bar = 10 mm. The muscle was expanded slightly to expose the detailed intramuscular innervation pattern. Muscular subbranches running within the muscle formed a characteristic tree-like branching pattern. The terminal plexus with tufty endings of muscular subbranches is visible near the muscle's proximal half. The muscular branch (MB) to the levator palpebrae superioris muscle reaches the superior rectus muscle's medial margin.

Three to six (mean = 4.6, median = 5, and SD = 0.88) primary branches arose from the superior branch of the oculomotor nerve; finally, based on Sihler's stain, from 4 to 8 (mean = 6.4, median = 7, and SD = 1.3) muscular subbranches were observed that reached the superior rectus muscle's internal surface (see Figure 7). The diameters of the primary branches to the superior rectus muscle ranged from 0.09 mm to 0.68 mm (mean = 0.25 mm, median = 0.19 mm, and SD = 0.15 mm). Measured from the muscle's insertion, the distance at which the motor nerve subbranches penetrated the superior rectus muscle's internal surface ranged from 20.5 mm to 33.4 mm (Table 2). This distance referenced (a quotient) to the muscle's total length ranged from 47.2% to 74.7% (Table 2). One or two branches from the superior branch of the oculomotor nerve wrapped around the superior rectus muscle's medial border and innervated the levator palpebrae superioris muscle; however, in 15 of 70 specimens (21.4%), a branch to the levator palpebrae superioris muscle pierced the superior rectus muscle.

The detailed intramuscular distribution of certain nerve subbranches was visualized using Sihler's stain (Figures 6 and 7). In both rectus muscles, a characteristic tree-like pattern of muscular subbranches' propagation was observed. Primary branches that reached both muscles split into secondary subbranches, which underwent numerous further branches. The most numerous terminal subbranches formed tufty branches and followed a course nearly parallel to the muscle's fibers. Territories of terminal subbranches covered parallel muscle strands, although the finest subbranches' endings appeared to overlap in adjacent areas. The location of the largest density of subbranches differed slightly for both muscles. The distance between the inferior rectus muscle's insertion and the nerves' subbranches' anterior-most terminations referenced (a quotient) to the muscle's total length ranged from 26.9% to 47.2% (Table 1). The same ratio calculated for the superior rectus ranged from 34.8% to 46.6% (Table 2).

## 4. Discussion

### 4.1. The Vertical Rectus Muscles' Anatomical Variations

Deviations from the typical eyeball muscle structure are rare. As Bergman et al. [7] and Kocabiyik [9] stated, occasionally, some of the four rectus muscles may be developed poorly or even absent. However, the literature suggests that such conditions are severe anomalies that are correlated with congenital craniofacial anomalies and cause significant clinical symptoms [24–27]. However, no specimens with craniofacial variations were available for our study, and we found no such severe alterations of the superior or inferior rectus muscle in our material.

Muscular slips may also link extraocular rectus muscles to each other, and different variants of those slips may be observed. Such variant muscular connections were found in our study in two of 70 cases (~2.8%). These findings are consistent with Bergman et al.'s [7] description, who stated that the superior rectus muscle may provide a muscular slip that also arises from the common tendinous ring and courses downward and forward across the optic nerve's lateral side to join the inferior rectus muscle. Occasionally, the muscular slips that originate from the common tendinous ring and attach to the rectus muscles are referred to as accessory rectus muscles. Topographically, the muscles from this group are located on the optic nerve's lateral side [2, 7, 9].

Disturbances in differentiating the superior and inferior ectomesenchymal complexes that may occur during early stages of embryological development may explain embryological typical muscular bridges between the superior and inferior rectus muscles [28]. There are only a few reports of muscular bridges or accessory rectus muscles, which vary with respect to the objects studied (cadavers or living subjects), clinical manifestation (absence or presence of ocular movement disorders), and the anatomical description's level of detail [8, 10, 29–35]. One of the most recent anatomical reports provided a complete anatomical description of bilateral accessory rectus muscles observed during the dissection of a 68-year-old male cadaver with no ocular movement abnormalities reported in the medical history [8]. In this case report, the accessory rectus muscle was divided into two “slips” or “bellies”—superior and inferior—located lateral to the optic nerve and the ophthalmic artery's main trunk. Both bellies originated with a short common tendon attached to the annulus of Zinn and were attached to the superior and inferior rectus muscles, respectively. These rare findings were recognized as the accessory (supernumerary) rectus muscles and resembled one of the cases described in our study (see Figure 4). Kakizaki et al. [30] reported a case of a variant muscle that linked the superior and inferior rectus muscles in the orbit of a 45-year-old female cadaver, whose medical history also indicated no ocular movement disorders. Kakizaki et al. [30] detected no definite nerve insertion in the accessory rectus muscles, as in both cases described in this report. Further, von Lüdinghausen [29] described a case of accessory rectus muscles' bilateral presentation found during dissection of an adult cadaver with no eye mobility problems. Thus, additional muscle bridges that connect the inferior and superior rectus muscles do not have to cause clinical symptoms and may be found incidentally during autopsy or diagnosis of unrelated visual symptoms. However, Kightlinger et al. [31] indicated that, “There is an increased incidence of orbital bands in patients with restrictive strabismus, eyeball retraction, and eyelid retraction.” According to Khitri and Demer [32], the frequency of various types of orbital bands in normal adult orthotropic subjects who underwent MRI of the orbit may be estimated at 0.8%, while this ratio is higher for patients with strabismus (2.4%). In the same study, the superior-inferior rectus bands were seen only in 33% of all types of bands detected within the orbit.

**Figure 8:**
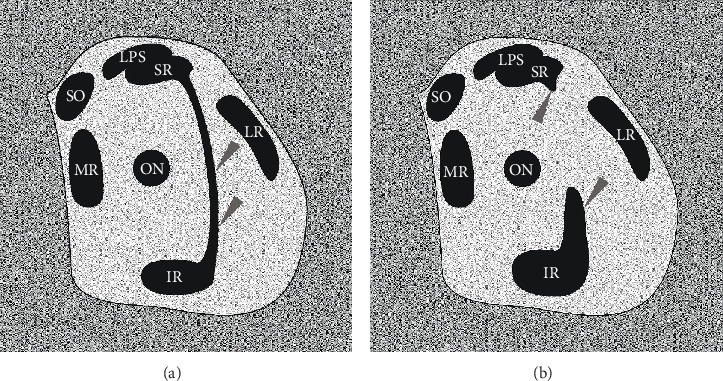
Schematic drawing simulating MRI or CT coronal scans that show the spatial organization of muscular structures within the orbit in the presence of atypical muscular slips that link the superior and inferior rectus muscles. The drawings were prepared on the basis of comparison of different MRI scans by Khitri and Demer (2010) and Kightlinger et al. (2017). (a) Complete muscular bridge seen between superior and inferior rectus muscles (marked by grey arrowheads). On drawing (b), only fragments of certain heads of the supernumerary rectus were captured (grey arrowheads). ir: inferior rectus muscle; lr: lateral rectus muscle; lps: levator palpebrae superioris muscle; mr: medial rectus muscle; sr: superior rectus; so: superior oblique muscle; on: optic nerve. This figure is a drawing derived from Haładaj et al. (2018) under the terms of the Creative Commons Attribution 4.0 International License (https://creativecommons.org/licenses/by/4.0/), which permits unrestricted use, distribution, and reproduction in any medium.

The occurrence of these variant muscular bands also has been observed during imaging studies. Orbital imaging has played an important role in extending our knowledge of the extraocular muscles and their associated connective tissues' structure and function [33, 34]. Kightlinger et al. [31] described a series of seven cases with muscular bands that linked the superior and inferior rectus muscles' lateral borders. According to the authors, the clinical meaning of muscular bands or slips located within the orbit “…is uncertain and possibly depends on size and location.” The drawings prepared based on the comparison of different MRI scans presented by Kightlinger et al. [31] and Khitri and Demer [32] are shown in Figure 8. During orbital imaging, variant orbital bands or bridges may be confused with blood vessels (arteries or veins) or with numerous pathologies that may be found within the orbit, such as lymphoma, orbital pseudotumor, vascular malformations, sarcoid, or metastases [8, 31]. Accessory muscular bands located within the orbit also may be correlated with ocular congenital cranial dysinnervation disorders, Gomez-Lopez-Hernandez syndrome, and Duane syndrome [31, 32, 35, 36]. Therefore, knowledge of normal anatomy and vertical rectus muscles' variations may be clinically relevant during orbital imaging and differential diagnoses of eye movement disorders and in surgical procedures involving this group of muscles [9, 29, 31, 32, 34, 37–39]. Demer et al. [34] concluded that extraocular muscles' and cranial nerves' imaging “…can provide important information in patients with strabismus” and may support definitive management decisions in many cases. Summing up, knowledge about the connective muscle slips between muscles may help for correct interpretations of MRI scans of the orbits. It may also be useful for planning ophthalmologic surgeries.

Other variations of the superior and inferior rectus muscles are rare. Nayak et al. [40] observed a unique case of a double-bellied superior rectus muscle in an adult male cadaver. Occasionally, a muscular branch running to the levator palpebrae superioris muscle may pierce the superior rectus muscle. Djordjević et al. [41] observed that the muscular branch to the levator palpebrae superioris muscle ran along the superior rectus muscle's medial border in seven of eight specimens (87.5%), while in one (12.5%), it passed through the superior rectus muscle and then entered the levator's inferior surface. In our study, we found branches that pierced the superior rectus muscle in 15 of 70 specimens (21.4%). Further, Bye et al. [42] confirmed that the muscular branch to the levator palpebrae superioris may pierce the superior rectus muscle. Bergman et al. [7] also stated that some of the oculomotor nerve's branches may pierce the inferior rectus muscle. Black et al. [43] stressed that the nerve to the inferior oblique muscle may be susceptible to injury when it travels along the inferior rectus muscle's lateral border. These anatomical relations between the nerve and inferior rectus muscle are crucial, as the inferior rectus muscle's traction may cause paresis of the inferior oblique muscle and also may trigger tonic pupil attributable to damage of parasympathetic fibers that course most commonly with fibers destined for the inferior oblique muscle [43]. Thus, surgeons must be particularly careful when performing procedures in this area during orbital fracture repair. When the nerve to the inferior oblique pierces the inferior rectus muscle, its fixation through the muscular branches appears stronger (in those cases, muscular branches to the inferior rectus muscle arise directly from the nerve to the inferior oblique muscle), which may increase the susceptibility to injury when the muscle is stretched [43].

Extraocular muscles also may show variations in size and attachments, both between different individuals and between particular muscles in this group. Stärk and Kuck [44] provided data on the average distance of all rectus muscles' anatomical insertion from the limbus. According to these data, the superior rectus muscle seems to be farther from the limbus (mean 6.64 mm) than is the inferior rectus muscle (mean 5.67 mm). This observation is consistent with our findings. However, our results (mean distance from the limbus was 6.2 mm for the inferior and 7.6 mm for the superior rectus muscle) are more similar to data provided by Shumway et al. [4, 5], who indicated that the inferior rectus muscle inserts 6.5 mm from the limbus and the superior rectus muscle 7.7 mm from the limbus. According to the authors, the inferior rectus muscle is 9.8 mm wide at its insertion on the eyeball and the muscle's entire length is 40 mm, while the superior rectus muscle's insertion is 10.6 mm wide and its entire length is 41.8 mm. In our study, the inferior rectus muscle's length ranged from 39.2 mm to 44.9 mm (mean = 41.8 mm) and its insertion ranged from 7.2 mm to 9.6 mm (mean = 8.2 mm), while the superior rectus muscle's length ranged from 41.2 mm to 49.3 mm (mean = 43.7 mm) and its insertion ranged from 8.1 mm to 11.5 mm (mean = 9.6 mm).

### 4.2. Superior and Inferior Rectus Muscles' Innervation Pattern

Only limited data are available on extraocular muscles' intramuscular nerve distribution. Gözil et al. [17] examined the branching patterns in rabbit oculomotor and trochlear nerves demonstrated by Sihler's stain technique and concluded that Sihler's whole mount nerve staining technique could be useful for detailed studies of the extraocular muscles' motor control. Based on their staining results, Gülekon et al. [18] observed more complex communications and a branching pattern in rabbits' superior oblique and superior rectus muscles. However, based on the results presented in this study and on other reports based on human material [2, 11, 13, 14, 21], we found that, apart from some subtle differences, the four rectus muscles share a common general pattern of intramuscular nerve subbranches' arborization, with characteristic Y-shaped ramifications that form the terminal nerve plexus located near half the muscle's length. However, the location of muscular subbranches' terminal endings seems to vary slightly among the muscles and the mean distance between the muscle's insertion. The nerves' subbranches' anterior-most terminations referenced (a quotient) to the muscle's total length may be estimated at 37.8% for the inferior rectus muscle, 40.9% for the superior rectus muscle, 43.3% (based on Haładaj et al. [21]) or 42.8% (based on Haładaj [23]) for the lateral rectus muscle, and 43.7% for the medial rectus muscle (based on [23]).

The extraocular muscles' large ratio of nerve fibers to skeletal muscle fibers gives these muscles excellent control and precision, in which the ratio is 1 : 3 to 1 : 5 compared to other skeletal muscles, which is 1 : 50 to 1 : 125 [4, 5]. The muscles' specific innervation pattern may allow selective activation of certain muscle regions during different movements or different stages of the eyeball's specific movement [12, 45]. According to Shin et al. [14], new information regarding the rectus muscles' nerve distribution pattern obtained by Sihler's stain may be helpful in understanding the muscles' function and strabismus' diverse pathophysiology. Detailed knowledge of these nerves' distribution also may help clarify physiological reflexes' complex mechanisms and eyeball movements' coordination. Clark and Demer [46] used magnetic resonance imaging in humans to investigate differential compartmental activity in the rectus extraocular muscles during head tilt, which evokes ocular counterrolling (a torsional vestibuloocular reflex). According to the authors, the vertical rectus extraocular muscles did not exhibit significant compartmental contractile changes during head tilt. However, vertical rectus muscles' secondary action is torsion: the superior rectus is involved in medial rotation, while the inferior rectus acts during lateral rotation [3, 4]. It seems likely that a specific distribution of muscular branches may promote selective activation of individual functional segments of the superior and inferior rectus muscles during torsion. However, further functional tests and observations are needed in this field.

## 5. Conclusions

Both the inferior and superior rectus muscles appear to demonstrate a relatively constant morphology. The superior rectus muscle is slightly longer, and its insertion is farther from the limbus of the cornea than is the inferior rectus muscle. The superior branch of the oculomotor nerve innervates the superior rectus muscle, while the inferior rectus muscle is innervated by the inferior branch of the oculomotor nerve or, occasionally, is innervated directly by the nerve to the inferior oblique muscle. The four rectus muscles share the common general pattern of intramuscular nerve subbranches' arborization, with characteristic Y-shaped ramifications that form the terminal nerve plexus located near half of the muscle's length. The extraocular muscles and nerves' unexpected anatomical variations may be relevant during orbital imaging or various surgical and reconstructive procedures.

## 6. Limitations

Sihler's stain technique's strength is that it allows one to observe the finer nerve subbranches (without additional auxiliaries) unavailable to manual dissection. This technique visualizes detailed branching patterns of examined nerves. However, the technique's limitations should be considered when assessing the results. The image obtained with this method is a result of multiple layers that overlap in the gelatinized specimen's entire thickness [21]. Further, it does not distinguish between sensory and motor neurons in the specimen [16]. Thus, various techniques, including three-dimensional histological reconstructions and immunohistochemical techniques, also should be compared to the results of Sihler's stain to obtain full information on intramuscular nerves' distribution.

## Figures and Tables

**Table 1:** Descriptive statistics for the data collected based on the measurements of the inferior rectus muscle and its motor nerve branches.

Variable	Min (mm)	Max (mm)	Mean (mm)	Median (mm)	SD (mm)
1	39.2	44.9	41.8	41.2	2
2	3.5	6.6	4.5	4.4	0.7
3	6.8	9.6	7.9	7.8	0.8
4	7.2	9.6	8.2	7.8	0.8
5	2.3	4.8	3.1	2.8	0.7
6	21.5	29.6	24.2	23.6	2.2
	Min (%)	Max (%)	Mean (%)	Median (%)	SD (%)
7	49.4	71.3	56.8	56.2	5.5
8	30.4	47.2	37.8	36.9	4.6

1: the inferior rectus muscle's length measured between its origin and the distal tendon's insertion; 2: origin's width; 3: muscle's width at midlength; 4: muscle's insertion width; 5: muscle's thickness at midlength; 6: distance at which the motor nerve subbranches penetrated the internal muscle's surface measured from the muscle's insertion; 7: distance between the muscle's insertion and the location where the oculomotor nerve's muscular subbranches penetrated the muscle's internal surface referenced (a quotient) to the muscle's total length expressed as a percentage; 8: distance between the muscle's insertion and the anterior-most terminations of the nerves' subbranches referenced (a quotient) to the muscle's total length.

**Table 2:** Descriptive statistics for the data collected based on the measurements of the superior rectus muscle and its motor nerve branches.

Variable	Min (mm)	Max (mm)	Mean (mm)	Median (mm)	SD (mm)
1	41.2	49.3	43.7	42.9	2.5
2	3.7	5.4	4.6	4.7	0.6
3	6.1	10.9	8.1	8.4	1.6
4	8.1	11.5	9.6	9.5	1.1
5	1.8	3.1	2.5	2.6	0.4
6	20.5	33.4	24.6	23.2	3.7
	Min (%)	Max (%)	Mean (%)	Median (%)	SD (%)
7	47.2	74.7	58.2	56	8
8	34.8	46.6	40.9	40.8	3.2

1: the superior rectus muscle's length measured between its origin and the distal tendon's end; 2: origin's width; 3: muscle's width at midlength; 4: muscle's insertion width; 5: muscle's thickness at midlength; 6: distance at which the motor nerve subbranches penetrated the internal muscle's surface measured from the muscle's insertion; 7: distance between the muscle's insertion and the location where the oculomotor nerve's muscular subbranches penetrated the muscle's internal surface referenced (a quotient) to the muscle's total length expressed as a percentage; 8: distance between the muscle's insertion and the anterior-most terminations of the nerves' subbranches referenced (a quotient) to the muscle's total length.

## Data Availability

The data used to support the findings of this study are available from the corresponding author upon request.
